# A systematic review and meta-analysis of healthcare service quality in Iran using the SERVQUAL model: a comparison before and after the Health Transformation Plan

**DOI:** 10.1186/s12913-026-14110-3

**Published:** 2026-04-02

**Authors:** Pouria Farrokhi, Ali Pezeshkian, Aidin Aryankhesal, Ehsan Zarei, Asgar Aghaei Hashjin, Morteza Arab‑Zozani, Hojjat Rahmani, Amirhossein Sahebkar

**Affiliations:** 1 Department of Health Management, Policy and Economics, School of Public Health, Tehran University of Medical Sciences, Tehran, Iran; 2 Department of Pharmacoeconomics and Pharmaceutical Administration, School of Pharmacy, Tehran University of Medical Sciences, Tehran, Iran; 3 Department of Health Services Management, School of Health Management and Information Sciences, Iran University of Medical Sciences, Tehran, Iran; 4 Department of Health Service Management, School of Public Health and Safety, Shahid Beheshti University of Medical Sciences, Tehran, Iran; 5 Health Management and Economics Research Center, Health Management Research Institute, Iran University of Medical Sciences, Tehran, Iran; 6 Social Determinants of Health Research Center, Birjand University of Medical Sciences, Birjand, Iran; 7 Biotechnology Research Center, Pharmaceutical Technology Institute, Mashhad University of Medical Sciences, Mashhad, Iran; 8 Centre for Research Impact & Outcome, Chitkara College of Pharmacy, Chitkara University, Rajpura, Punjab 140401 India; 9 Applied Biomedical Research Center, Basic Sciences Research Institute, Mashhad University of Medical Sciences, Mashhad, Iran

**Keywords:** Quality of health care, Patient satisfaction, Health care reform, Delivery of health care, Primary health care, SERVQUAL, Iran

## Abstract

**Background:**

The quality of service constitutes a crucial element in achieving patient satisfaction and population health. The Health Transformation Plan (HTP) represented a pivotal initiative within the Iranian health system, aimed at enhancing the quality of health services. Therefore, this study aimed to determine the effect of Iran’s HTP on the quality of health services based on the SERVQUAL model.

**Methods:**

This systematic review and meta-analysis was conducted in 2023, following the PRISMA guidelines. A comprehensive search was performed in national and international databases, including Web of Science, PubMed, Scopus, SID, and MagIran. The quality of the studies was assessed using the 22-item STROBE checklist. Overall expectation and overall perception scores were calculated as the pooled mean values across the five SERVQUAL dimensions. The data analysis was conducted using Comprehensive Meta-Analysis software.

**Results:**

A total of 51 studies, comprising a total sample size of 19,679, were included in the analysis. The pooled estimate of the mean for overall expectation before and after the HTP was 4.646 (95% CI: 4.48–4.81, *P* < 0.001) and 4.678 (95% CI: 4.29–5.06, *P* < 0.001), respectively. Moreover, the pooled estimate of the mean for overall perception before and after the HTP was 3.785 (95% CI: 3.56–4.01, *P* < 0.001) and 3.806 (95% CI: 3.53–4.07, *P* < 0.001), in that order. After the HTP, the highest and lowest gap was related to empathy (Gap = -0.9) and tangibles (Gap = -0.852), respectively. Additionally, there was a 0.176 decrease in the quality gap for inpatient services, a 0.02 increase for outpatient services, and a 0.24 rise for primary health care (PHC) services.

**Conclusion:**

Our study shows that although patients’ expectations and perceptions improved slightly after the HTP, the overall quality gap widened, with the greatest deterioration occurring in primary healthcare. These findings underscore the need for more balanced resource allocation, stronger support for the PHC workforce, and enhanced patient-centered practices, particularly communication, empathy, and responsiveness. Prioritizing these areas may help improve service quality across all levels of Iran’s health system.

## Background

Service quality is a crucial predictor of patient satisfaction and population health [1]. Global organizations such as the WHO, World Bank, and OECD have consistently prioritized service quality within the frameworks of universal health coverage (UHC) and the Sustainable Development Goals [2, 3]. It has been estimated that more than eight million individuals per year in low- and middle-income countries (LMICs) die from conditions that should, in principle, be treatable by the health system. Furthermore, the economic losses incurred by these deaths reached an estimated US$6 trillion in 2015 alone [4]. Given the complexity, cost, and safety risks inherent in healthcare delivery, ensuring high-quality services is essential [5, 6]. There is ample evidence from high-income countries demonstrating a clear relationship between health-service quality and health outcomes [7, 8]. Furthermore, there are discernible connections between initiatives aimed at enhancing the quality of health services and advancements towards UHC, the development of resilient health services as a foundation for health security, and the provision of services in fragile and vulnerable contexts [3].

As an LMIC, Iran has implemented substantial measures to enhance the quality of its healthcare services, achieve UHC, and improve the responsiveness of its health system. The most recent initiative is the Health Transformation Plan (HTP). Implemented in 2014, the HTP’s stated objectives are to enhance public access to healthcare services, improve service quality, and reduce out-of-pocket (OOP) expenses. In this regard, several measures have been implemented to improve the quality of hospital services, increase the number of specialists, and enhance hospital facilities [9, 10]. The latest research findings suggest that HTP has had a considerable impact on healthcare access, patient satisfaction, and insurance coverage [11, 12], the 2.5% reduction in OOP payments, along with a decline from 2.9% to 2.1% of the population facing catastrophic health expenditure from 2014 to 2015 [13].

Following nearly a decade of implementing HTP, the Iranian health system is confronted with a number of considerable challenges. These include the lack of financial sustainability [14], the potential bankruptcy of insurance organizations [15, 16], insufficient governance and leadership [15], and issues related to the availability and retention of specialized and permanent healthcare personnel [14, 17]. Collectively, these challenges have the potential to negatively impact the accessibility, efficiency, and quality of care.

The monitoring and evaluation of healthcare services enables the identification of potential improvements, more effective resource allocation, and assurance of optimal patient care [18, 19]. In order to assess the quality of healthcare services, a series of foundational models and instruments have been developed by Gronroos in 1984 [20], Parasuraman et al. in 1985 [21], Haywood-Farmer in 1988 [22], Donabedian in 1988 [23], Brogowicz et al. in 1990 [24], and Cronin & Taylor in 1992 [25]. The SERVQUAL model, developed by Parasuraman et al., is a widely used approach for assessing healthcare service quality and conceptualizes quality as the gap between patients’ expectations and perceptions across five domains. In the SERVQUAL framework, assurance reflects staff competence and their ability to inspire trust; tangibles capture the appearance of physical facilities, equipment, and personnel; reliability refers to the consistent and dependable delivery of promised services; responsiveness denotes the willingness and promptness of staff in helping; and empathy encompasses individualized, compassionate care. SERVQUAL operationalizes these domains through multi-item Likert-scale questionnaires that measure both patients’ expectations and their perceived experiences, and the quality gap for each domain is calculated as the difference between perception and expectation [26–28]. In Iran, numerous researchers have employed this instrument to assess the quality of health services, and it has gained considerable acceptance [29, 30].

To inform evidence-based decision-making, policymakers and health managers must understand how the quality of healthcare services has evolved across major reforms such as the HTP. Although the SERVQUAL model has been widely applied in Iran, previous studies and reviews have not provided an integrated assessment of service quality across inpatient, outpatient, and primary healthcare settings or examined temporal changes before and after the HTP. Moreover, no meta-analysis has evaluated how mean scores and quality gaps across SERVQUAL dimensions shifted following the reform, despite ongoing concerns about patient satisfaction and service quality. By addressing these gaps, the present study offers the most comprehensive system-wide evaluation of healthcare service quality associated with the HTP, using the SERVQUAL model as the analytical framework.

## Method

In 2023, the present study was conducted following the PRISMA guidelines [31]. The review protocol was developed before data extraction but was not registered in any public registry. The procedures entailed the following stages: identification, screening, eligibility assessment, and selection of studies. A third reviewer (EZ) was consulted at each stage in the process if consensus could not be reached.

### Identification process

A comprehensive search of national and international databases, including Web of Science, PubMed, Scopus, MagIran, and the Scientific Information Database (SID), was conducted to identify relevant publications. The Medical Subject Headings (MeSH) system was utilized to facilitate the retrieval of pertinent literature, with the following search terms employed: “Quality of health care”, “Service quality”, “Quality of service”, “Health quality”, SERVQUAL, “Patient^*^ perception”, “Patient^*^ expectation”, Perception, Expectation, and Iran. The time restriction was applied from 2007 to 2022. This timeframe was selected to allow for a balanced comparison of perceived and expected service quality across two equal eight-year periods before and after the HTP, ensuring that trends in service quality could be meaningfully analyzed. Table 1 presents the comprehensive search strategy employed in PubMed. Grey literature screening resources, including conference papers and the Google Scholar database, were incorporated into these searches.

**Table 1:** The search strategy employed in PubMed was adapted for use in other databases

Database	Set	Search Strategy		Records (No)
PubMed	#1	MeSH	“Quality of health care”	8,461,365
		Title & Abstract	“Quality of health care” OR “Service quality” OR “Quality of service” OR “Health quality”	
	#2	Title & Abstract	SERVQUAL OR “Patient perception” OR “Patient expectation” OR “Gap analysis” OR Perception OR Expectation	274,98
	#3	MeSH	Iran	70,885
		Title & Abstract	Iran	
	#4		#1 AND #2 AND #3	461
	#5		Filters: from 2007–2022	394

### Screening process

In accordance with PRISMA guidelines, the study employed a rigorous screening process to identify relevant literature. Duplicate records were eliminated, and the remaining records were exported to Endnote X9 for further examination. Following independent abstract and title screenings, two reviewers (PF & AP) identified potential papers for further analysis and data extraction.

### Inclusion criteria

The following criteria were employed in the selection of the studies: (1) Original studies; (2) Articles using the SERVQUAL instrument; (3) Studies that provided the mean score for service quality dimensions from the patients’ viewpoint; (4) The availability of full-text articles; (5) English and Persian-language articles; (6) Articles published from 2007 to 2022. Studies conducted eight years before (from 2007 to 2014) and after (from 2015 to 2022) the HTP were reviewed. In the process of data entry, the year in which the study was conducted was considered rather than the publication year.

To ensure methodological consistency, only studies employing a quantitative design based on a Likert-scale SERVQUAL questionnaire were included. Qualitative studies were excluded because they do not provide numerical mean scores required for quantitative synthesis and meta-analysis. Additionally, studies that solely presented employee viewpoints, failed to include portions of the mean score, did not employ a Likert scale to assess service quality, or lacked sufficient quality were excluded.

### Study selection

Two authors (PF and AP) independently reviewed the full texts of all the included studies. The third author (EZ) carried out a further review of all eligible or potentially eligible studies. Data extraction was also performed independently by the same two reviewers, and discrepancies were resolved by a third reviewer.

The quality of the studies was also assessed using the 22-item Strengthening the Reporting of Observational Studies in Epidemiology (STROBE) checklist [32]. A score of 0–7 indicates poor, 8–17 moderate, and 18–22 good quality. A standard data collection form was used to collect data on author(s), year of study, study language, setting, sample size, and mean scores of service quality dimensions.

### Synthesis methods

A random-effects model of meta-analysis was conducted using the Mantel-Haenszel method to assess overall perception and expectation [33]. All data were entered into the comprehensive meta-analysis (CMA) software version 3 in terms of mean and standard deviation (SD), and the mean and standard error with 95% confidence interval (CI) were reported for each variable. The extent of heterogeneity was evaluated through the use of the I-squared (I²) statistic test. The potential for publication bias was assessed using Egger’s test and visual inspection of the funnel plot.

Due to the considerable heterogeneity between the included studies with regard to variables such as time, setting, and design, we conducted a series of subgroup analyses based on the data, including comparisons before and after the implementation of the HTP, different settings (inpatients, outpatients, and PHC), and different dimensions (tangibles, reliability, responsiveness, assurance, and empathy). This approach was employed to address the issue mentioned above. In addition to subgroup analyses, a random-effects model was applied to account for methodological and contextual variability across studies. The high heterogeneity primarily reflected differences in study populations, settings, and assessment periods. Although meta-regression was considered to further explore the sources of heterogeneity, it was not feasible due to insufficient reporting of key moderator variables in the included studies.

## Results

**Fig. 1:**
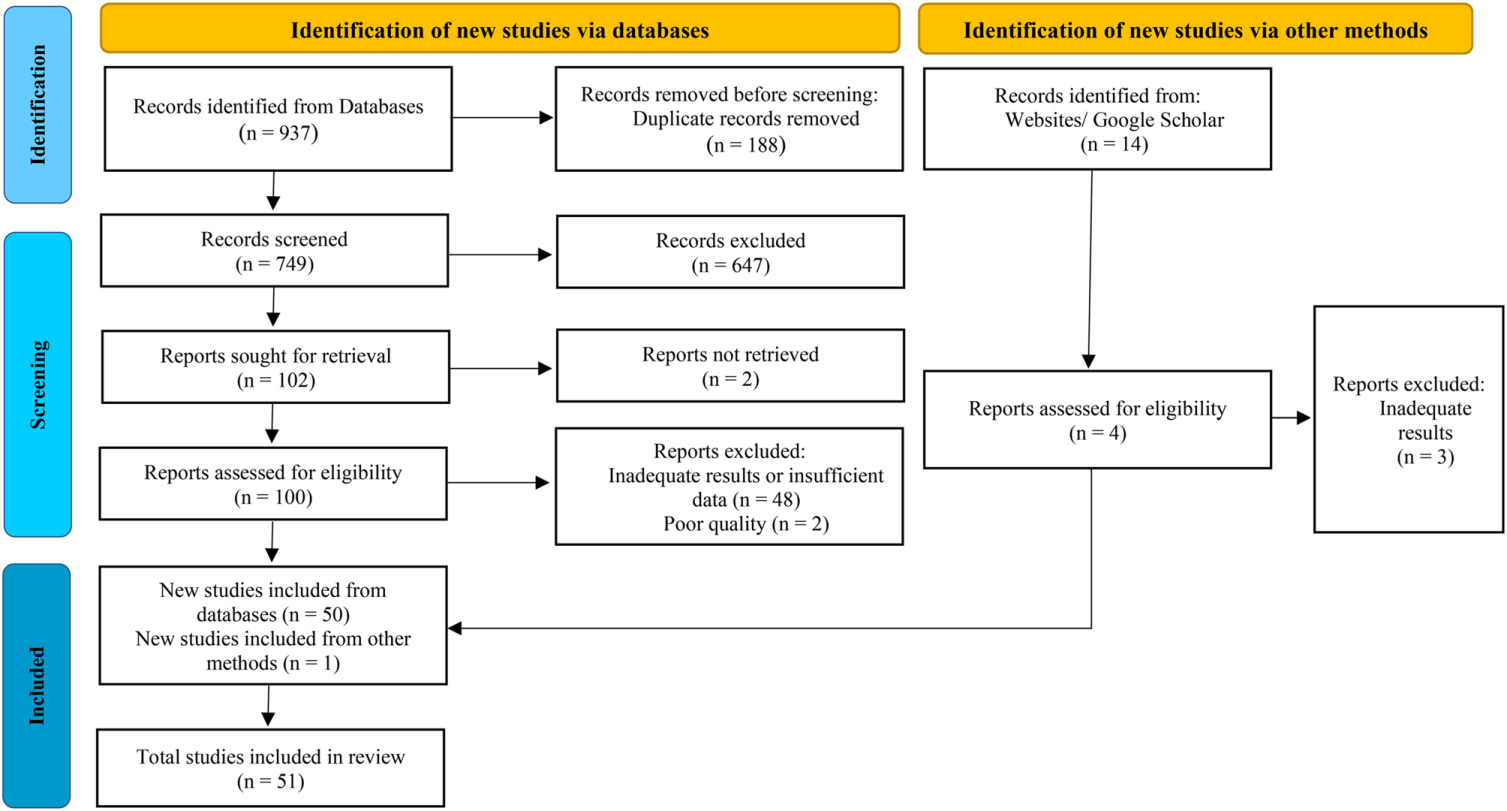
PRISMA 2020 flow diagram of the study selection process

The PRISMA diagram outlines the selection process, showing that from 937 database records and 14 additional sources, duplicates and ineligible reports were removed, resulting in a final inclusion of 51 studies (Fig. 1). Some full-text articles were excluded because essential quantitative data were not reported, including studies that did not report mean SERVQUAL scores [34], reported only quality gaps [35], omitted expectation scores [36], or assessed service quality from the perspective of providers (staff) rather than patients [37]. The majority of the articles were related to the period before the HTP (*n* = 30). Most of the studies were conducted in Tehran (*n* = 10) and in 2015 (*n* = 9). Nearly 57% of the studies were published in Persian. The total number of participants was 19,679 and the minimum and maximum sample size varied between 96 and 1,920. Based on the STROBE assessment, 41 studies (80.4%) were rated as good and 10 studies (19.6%) as moderate in methodological quality. The quality of inpatient, PHC, and outpatient services was assessed in 23, 16, and 12 studies, respectively. For the dimensions of empathy (*n* = 23) and assurance (*n* = 27), most studies reported the lowest and highest mean perceptions, respectively (Table 2).

**Table 2:** A synthesis of the selected studies on the measurement of health service quality

	Author(s)	Year	Location	Sample size	Setting	The lowest perception mean score	The highest perception mean score	Quality score
Before the HTP *n* = 30	Mohammadi & Shoghli [38]	2007	Zanjan	300	PHC	Empathy	Tangibles	Moderate
	Ghanbari et al. [39]	2007	Tehran	500	PHC	Tangibles	Assurance	Good
	Aghamolaei et al. [40]	2008	Bandar Abbas	400	PHC	Empathy	Assurance	Good
	Nekoei-Moghadam & Amiresmaili [41]	2008	Kerman	385	Inpatient	Tangibles	Assurance	Good
	Tabibi et al. [42]	2009	Tehran	242	Outpatient	Reliability	Tangibles	Good
	Mohammadi & Mohammadi [43]	2009	Zanjan	300	PHC	Responsiveness	Tangibles	Good
	Hekmatpo et al. [44]	2010	Tehran	260	Inpatient	Empathy	Assurance	Good
	Jenaabadi et al. [45]	2010	Zahedan	200	Inpatient	Empathy	Tangibles	Moderate
	Havasbeigi et al. [46]	2010	Ilam & Kermanshah	450	Outpatient	Responsiveness	Tangibles	Moderate
	Tarrahi et al. [47]	2010	Khorramabad	650	PHC	Empathy	Reliability	Good
	Gholami et al. [48]	2010	Neyshabour	400	PHC	Tangibles	Assurance	Good
	Zarei et al. [49]	2010	Tehran	983	Inpatient	Empathy	Tangibles	Good
	Gorji & Akbari [50]	2011	Tehran	116	Inpatient	Tangibles	Assurance	Moderate
	Ajam et al. [51]	2011	Zabol	100	Inpatient	Tangibles	Reliability	Good
	Vafaee-Najar et al. [52]	2012	Mashhad	480	PHC	Responsiveness	Assurance	Good
	Razlansari et al. [53]	2012	Kermanshah	400	Inpatient	Reliability	Empathy	Good
	Ameryoun et al. [54]	2012	Tehran	264	Inpatient	Empathy	Assurance	Good
	Nabilou & Rasouli [55]	2012	West Azerbaijan	390	PHC	Responsiveness	Assurance	Good
	Naqavi et al. [56]	2012	Kerman	260	Outpatient	Assurance	Reliability	Good
	Ghobadi et al. [57]	2013	Ardebil	650	Outpatient	Empathy	Assurance	Moderate
	Gholami et al. [58]	2013	Shiraz	200	Inpatient	Tangibles	Assurance	Good
	Dopeykar et al. [59]	2013	Tehran	385	Outpatient	Empathy	Assurance	Good
	Sina & Nadi [60]	2013	Sari	331	Inpatient	Assurance	Empathy	Good
	Ayoubian et al. [61]	2013	Isfahan	104	Inpatient	Tangibles	Assurance	Good
	Aghamolaei et al. [62]	2013	Bandar Abbas	96	Inpatient	Responsiveness	Assurance	Good
	Bahadori et al. [63]	2014	Kerman	195	Inpatient	Empathy	Assurance	Good
	Bastani et al. [64]	2014	Shiraz	200	Outpatient	Responsiveness	Assurance	Good
	Alijanzadeh et al. [65]	2014	Qazvin	1002	Inpatient	Empathy	Assurance	Good
	Khaki et al. [66]	2014	Shiraz	400	Outpatient	Empathy	Reliability	Good
	Kazemnezhad et al. [67]	2014	Qom	409	PHC	Empathy	Assurance	Good
**After the HTP** *n* = 21	Rezaei et al. [68]	2015	Kermanshah	400	Inpatient	Reliability	Responsiveness	Good
	Qolipour et al. [69]	2015	Ahvaz	250	Inpatient	Responsiveness	Tangibles	Good
	Adhami et al. [70]	2015	Tehran	405	Inpatient	Tangibles	Assurance	Moderate
	Razmjoee et al. [71]	2015	Shiraz	98	Inpatient	Assurance	Reliability	Good
	Haghshenas et al. [72]	2015	Tehran	225	Outpatient	Responsiveness	Tangibles	Good
	Gholami et al. [73]	2015	Shiraz	100	Inpatient	Responsiveness	Assurance	Moderate
	Torabipour et al. [74]	2015	Ahvaz	255	Inpatient	Tangibles	Assurance	Good
	Karami Matin et al. [75]	2015	Kermanshah	400	PHC	Responsiveness	Assurance	Good
	Bahmei et al. [76]	2015	Shiraz	582	Inpatient	Empathy	Tangibles	Good
	Vizvari et al. [77]	2016	Gorgan	175	Inpatient	Responsiveness	Tangibles	Good
	Kashfi et al. [78]	2016	Ahvaz	384	PHC	Empathy	Reliability	Good
	Gholipour et al. [79]	2018	Sanandaj	384	PHC	Empathy	Tangibles	Moderate
	Gholipour et al. [80]	2018	Sanandaj	1920	PHC	Empathy	Tangibles	Moderate
	Kabir et al. [81]	2019	Golestan	384	Outpatient	Empathy	Assurance	Good
	Sharifi et al. [82]	2019	Mashhad	200	PHC	Empathy	Tangibles	Good
	Farrokhi et al. [83]	2019	Tehran	433	Outpatient	Responsiveness	Assurance	Good
	Kashfi et al. [84]	2019	Shiraz	300	Outpatient	Empathy	Assurance	Good
	Cheraghi et al. [85]	2020	Hamadan	701	PHC	Empathy	Assurance	Good
	Joulaei et al. [86]	2020	Shiraz	299	Outpatient	Empathy	Reliability	Good
	Danehchin et al. [87]	2020	Ahvaz	332	Inpatient	Empathy	Tangibles	Good
	Bahmani et al. [88]	2021	Sanandaj	400	PHC	Responsiveness	Assurance	Moderate

**Fig. 2:**
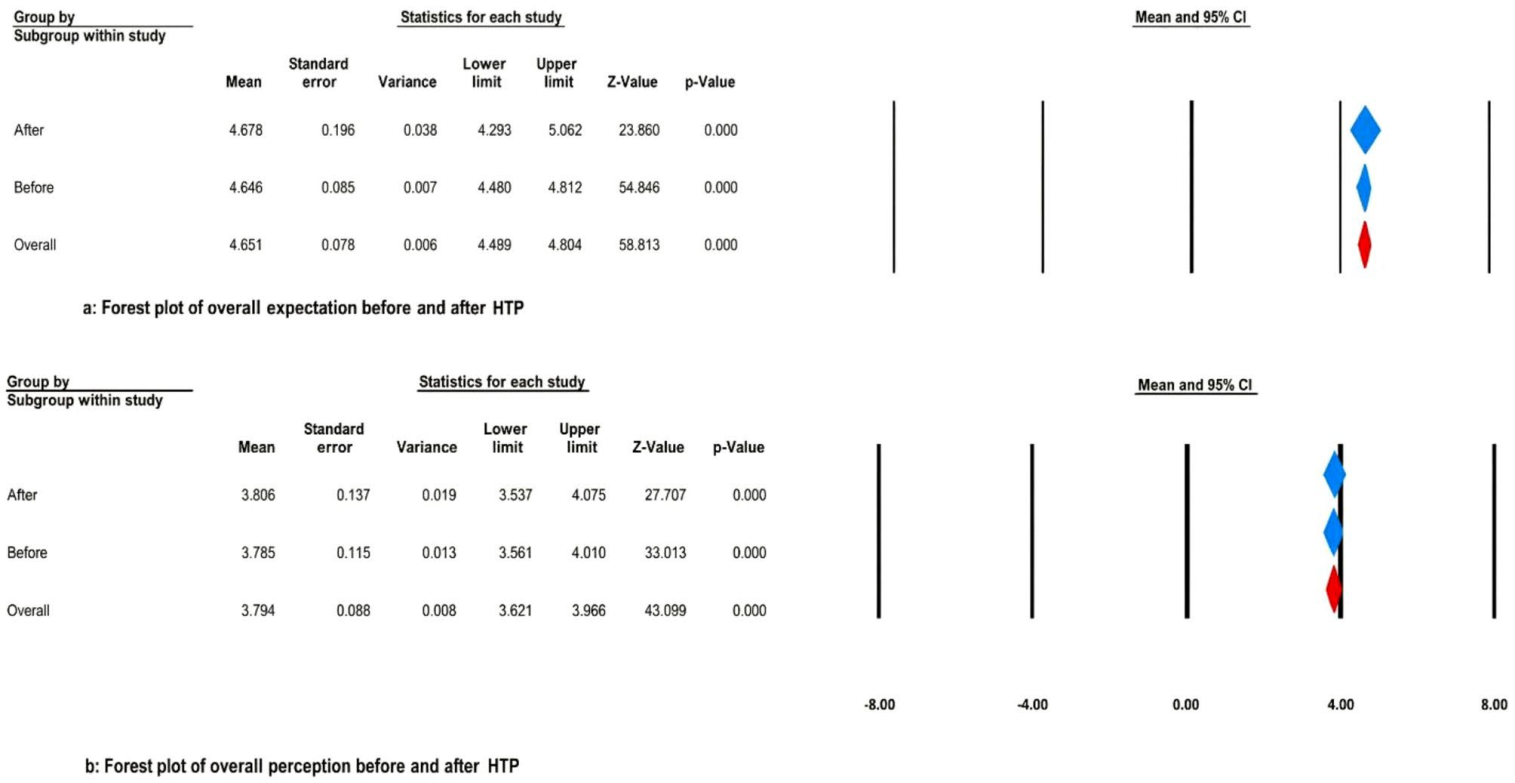
Forest plot of overall perception before and after HTP

**Fig. 3:**
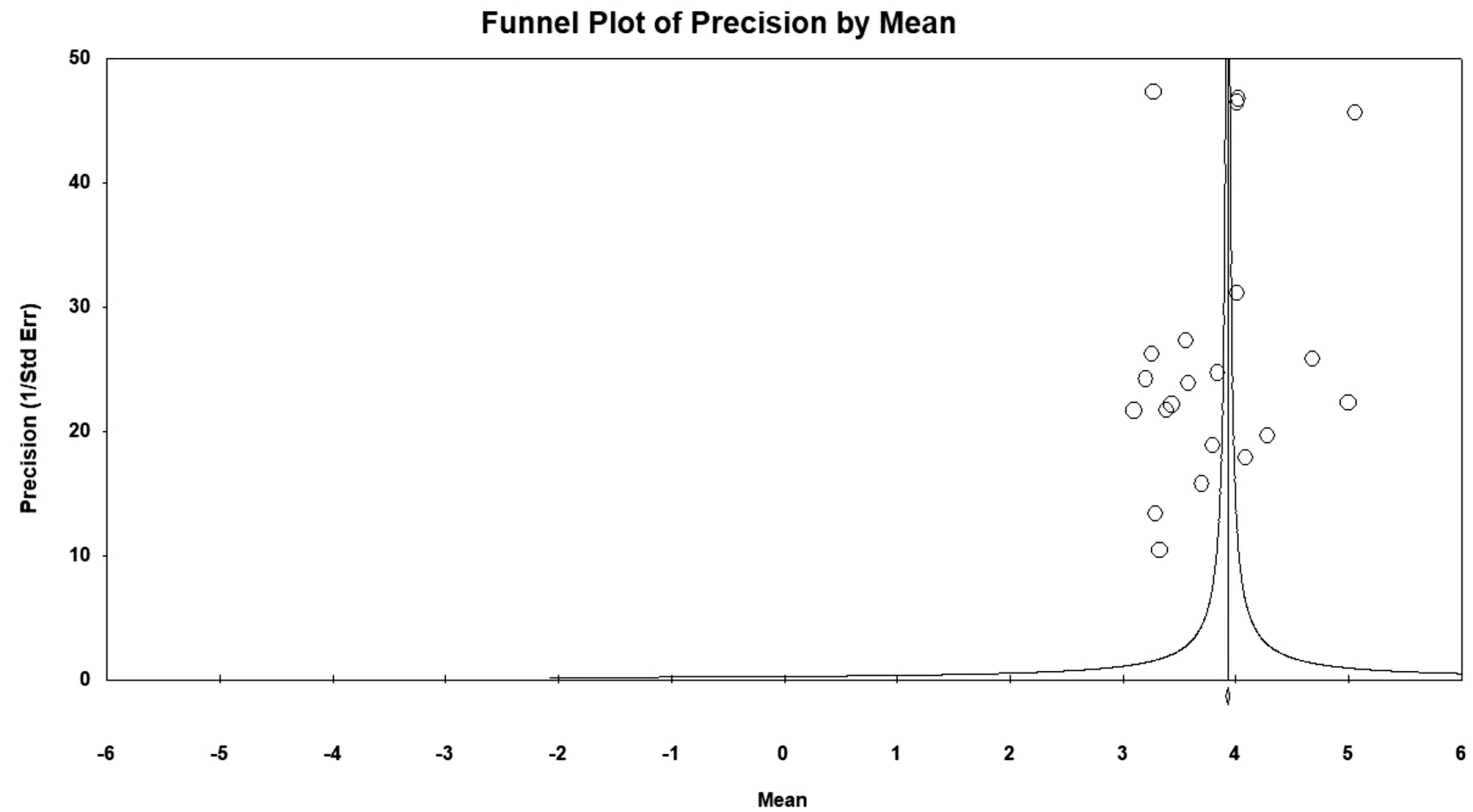
Funnel plot of publication bias for mean differences in quality of health care services

The pooled estimate of the mean for overall expectation before and after the HTP was, by the random effect model, 4.646 (95% CI: 4.48–4.81, *P* < 0.001) and 4.678 (95% CI: 4.29–5.06, *P* < 0.001), respectively (Fig. 2-a). Furthermore, as shown in Fig. 2-b, the pooled estimate of the mean for overall perception before and after the HTP was 3.785 (95% CI: 3.56–4.01, *P* < 0.001) and 3.806 (95% CI: 3.53–4.07, *P* < 0.001), respectively. As a result, the gap in the quality of health services has increased from − 0.861 (before the HTP) to -0.872 (after the HTP). In addition, as Fig. 3 demonstrates, the funnel plot’s visual inspection did not show any evidence of publication bias. Also, Egger’s test results confirmed this claim (*P* = 0.296).

Before the HTP, the highest and lowest pooled estimate of perception mean scores were related to assurance (mean = 3.87, 95% CI: 3.65–4.1, *P* < 0.001), and empathy (mean = 3.69, 95% CI: 3.5–3.88, *P* < 0.001), respectively. Also, the highest and lowest pooled estimate of expectation mean scores were related to reliability (mean = 4.7, 95% CI: 4.53–4.87, *P* < 0.001), and empathy (mean = 4.59, 95% CI: 4.37–4.8, *P* < 0.001). In addition, the highest and lowest gap was related to responsiveness (Gap= -0.935), and assurance (Gap= -0.81), in that order.

After the HTP, the highest and lowest pooled estimate of perception mean scores were related to assurance (mean = 4.01, 95% CI: 3.71–4.32, *P* < 0.001), and responsiveness (mean = 3.54, 95% CI: 3.22–3.86, *P* < 0.001), respectively. Moreover, the highest and lowest pooled estimate of expectation mean scores were related to assurance (mean = 4.88, 95% CI: 4.45–5.3, *P* < 0.001), and responsiveness (mean = 4.4, 95% CI: 4.01–4.8, *P* < 0.001). Additionally, the highest and lowest gap was related to empathy (Gap= -0.9), and tangibles (Gap= -0.852), respectively. Ultimately, the most significant decline in the service quality gap was observed in the responsiveness dimension, which decreased from 0.935 (before the HTP) to 0.863 (after the HTP) (Table 3).

**Table 3:** Subgroup analysis based on the dimensions

Dimensions			Number of studies	Mean	Gap (P-E)*	SE	95% CI		Heterogeneity	
							Lower	Upper	I^2^	*P*-value
Before the HTP	Perception	Tangibles	30	3.764	-0.856	0.08	3.607	3.922	99.464	*P* < 0.001
		Reliability	30	3.802	-0.902	0.104	3.598	4.005	99.671	*P* < 0.001
		Responsiveness	30	3.723	-0.935	0.105	3.517	3.929	99.652	*P* < 0.001
		Assurance	30	3.877	-0.81	0.114	3.653	4.101	99.737	*P* < 0.001
		Empathy	30	3.692	-0.899	0.097	3.503	3.882	99.576	*P* < 0.001
	Expectation	Tangibles	30	4.62	-	0.08	4.463	4.778	99.926	*P* < 0.001
		Reliability	30	4.704	-	0.087	4.534	4.874	99.915	*P* < 0.001
		Responsiveness	30	4.658	-	0.082	4.498	4.818	99.89	*P* < 0.001
		Assurance	30	4.687	-	0.081	4.528	4.847	99.89	*P* < 0.001
		Empathy	30	4.591	-	0.11	4.376	4.807	99.919	*P* < 0.001
After the HTP	Perception	Tangibles	21	3.981	-0.852	0.16	3.667	4.295	99.719	*P* < 0.001
		Reliability	21	3.947	-0.887	0.17	3.614	4.281	99.75	*P* < 0.001
		Responsiveness	21	3.545	-0.863	0.163	3.225	3.865	99.673	*P* < 0.001
		Assurance	21	4.018	-0.863	0.155	3.713	4.322	99.685	*P* < 0.001
		Empathy	21	3.564	-0.9	0.145	3.28	3.849	99.607	*P* < 0.001
	Expectation	Tangibles	21	4.833	-	0.213	4.416	5.251	99.949	*P* < 0.001
		Reliability	21	4.834	-	0.207	4.428	5.24	99.94	*P* < 0.001
		Responsiveness	21	4.408	-	0.202	4.012	4.804	99.936	*P* < 0.001
		Assurance	21	4.881	-	0.218	4.455	5.308	99.949	*P* < 0.001
		Empathy	21	4.464	-	0.21	4.053	4.875	99.944	*P* < 0.001

*Gap= Perception mean score – Expectation mean score

As indicated in Table 4, before the HTP, the mean score of perception in inpatient, outpatient, and PHC settings was 3.9 (95% CI: 3.54–4.27, *P* < 0.001), 3.58 (95% CI: 3.54–4.27, *P* < 0.001), 3.75 (95% CI: 3.32–4.17, *P* < 0.001), respectively. Also, the mean score of expectation in inpatient, outpatient, and PHC settings was 4.76 (95% CI: 4.48–5.05, *P* < 0.001), 4.34 (95% CI: 4.06–4.63, *P* < 0.001), and 4.69 (95% CI: 4.47–4.91, *P* < 0.001), in that order. Furthermore, the gap in inpatient, outpatient, and PHC was − 0.858, -0.762, and − 0.941, respectively.

After the HTP, the mean score of perception in inpatient, outpatient, and PHC settings was 3.56 (95% CI: 3.35–3.77, *P* < 0.001), 3.58 (95% CI: 3.2–3.95, *P* < 0.001), 4.27 (95% CI: 3.77–4.78, *P* < 0.001), respectively. Additionally, the mean score of expectation in inpatient, outpatient, and PHC settings was 4.24 (95% CI: 3.92–4.56, *P* < 0.001), 4.36 (95% CI: 4.04–4.69, *P* < 0.001), and 5.45 (95% CI: 4.66–6.25, *P* < 0.001), in that order. Also, the gap in inpatient, outpatient, and PHC services was − 0682, -0.782, and − 1.181, respectively. Following the HTP’s beginning, there has been a 0.176 reduction in the quality gap for inpatient services, a 0.02 rise for outpatient services, and a 0.24 increase for PHC services.

**Table 4:** Subgroup analysis based on the settings

Settings			Number of studies	Mean	Gap (P-E)	SE	95% CI		Heterogeneity	
							Lower	Upper	I^2^	*P*-value
Before the HTP	Perception	Inpatient	14	3.908	-0.858	0.186	3.543	4.272	99.885	*P* < 0.001
		Outpatient	7	3.586	-0.762	0.166	3.261	3.911	99.376	*P* < 0.001
		PHC	9	3.75	-0.941	0.216	3.327	4.174	99.748	*P* < 0.001
	Expectation	Inpatient	14	4.766	-	0.146	4.48	5.053	99.945	*P* < 0.001
		Outpatient	7	4.348	-	0.146	4.063	4.633	99.77	*P* < 0.001
		PHC	9	4.691	-	0.113	4.47	4.913	99.808	*P* < 0.001
After the HTP	Perception	Inpatient	9	3.562	-0.682	0.107	3.352	3.773	98.037	*P* < 0.001
		Outpatient	5	3.584	-0.782	0.191	3.209	3.959	99.355	*P* < 0.001
		PHC	7	4.278	-1.181	0.258	3.772	4.784	99.766	*P* < 0.001
	Expectation	Inpatient	9	4.244	-	0.164	3.923	4.565	99.593	*P* < 0.001
		Outpatient	5	4.366	-	0.166	4.041	4.69	99.618	*P* < 0.001
		PHC	7	5.459	-	0.407	4.661	6.257	99.974	*P* < 0.001

## Discussion

The findings of this study indicated a slight increase in both patients’ expectations and perceptions following the implementation of the HTP. Specifically, the pooled mean expectation increased from 4.646 (95% CI: 4.48–4.81, *P* < 0.001) before the HTP to 4.678 (95% CI: 4.29–5.06, *P* < 0.001) after its implementation. Similarly, the pooled mean perception rose slightly from 3.785 (95% CI: 3.56–4.01, *P* < 0.001) to 3.806 (95% CI: 3.53–4.07, *P* < 0.001). Despite these small changes, the overall quality gap widened from − 0.861 to − 0.872. These results suggest that although perceived service quality improved marginally, patients’ expectations grew at a higher rate, thereby exacerbating the overall service quality gap. In other words, the implementation of the HTP not only failed to reduce the gap between patients’ expectations and perceptions but may have contributed to its expansion. This trend is consistent with findings from other meta-analyses conducted in Iran. For instance, studies by Mohseni et al. [89], Rezaei et al. [30], Rahmani et al. [90] and Farrokhi et al. [1], reported significant gaps of -0.94, -0.90, -0.53 and − 0.48, respectively, indicating that patient expectations frequently exceed their actual perceptions of received care.

Before the implementation of the HTP, the highest and lowest service quality gaps were observed in the dimensions of responsiveness (Gap = -0.935) and assurance (Gap = -0.81), respectively. These findings suggest that, before the HTP, aspects such as timely service delivery, prompt attention to patient needs, and responsiveness to inquiries were not aligned with patient expectations. Consistent with these results, several previous studies have also identified responsiveness as the dimension with the most substantial gap [91–94]. Notably, within this dimension, the subcomponent related to providing timely services with minimal waiting time has been reported as the most significant contributor to the overall gap [95, 96]. In contrast, the assurance dimension appeared to be more favorable. This suggests that healthcare staff were relatively successful in demonstrating knowledge, courtesy, and the ability to instill confidence and trust in patients. Similar findings have been reported in other studies, which consistently identified assurance as the dimension with the smallest gap between expectations and perceptions [97–99].

After the HTP, the highest and lowest gap was related to empathy (Gap= -0.9), and tangibles (Gap= -0.852), respectively. Providers failed to address patient needs in a satisfactory manner, demonstrated inadequate concern for their patients’ wellbeing and failed to provide the necessary level of care. A primary objective of the HTP was to extend insurance coverage, augment the presence of specialists in government hospitals, and mitigate the financial burden on patients for inpatient care. This may have prompted a surge in individuals seeking treatment at government healthcare facilities, leading to an uptick in the workload of personnel and, consequently, a reduction in attention paid to patient needs and empathy. Following our findings, the AlOmari study revealed that the most significant negative gaps among the SERVQUAL items were associated with the listening skills of hospital staff and the amount of time spent with patients [27]. Previous studies conducted in Iran and China revealed the most significant discrepancy between expectations and perceptions with regard to the empathy dimension [100, 101]. Conversely, other studies indicated the smallest gap in this regard [29, 94, 95]. The empathy dimension has been identified as a key factor influencing patient satisfaction in previous studies, and is also a significant predictor of service quality [102, 103].

The tangibles dimension, which encompasses the physical environment, equipment, and staff appearance, was found to be among the most favorable, demonstrating the lowest gap between patient expectations and perceptions. Previous research has highlighted that tangible aspects of a healthcare setting form the basis of patients’ first impressions, with clean, well-maintained, and aesthetically pleasing environments positively influencing perceptions of service quality [104]. It is likely that improvements in the “hoteling” standards of public hospitals following the implementation of the HTP, including extensive renovations and refurbishment efforts, played a key role in narrowing the gap within this dimension [9, 10]. Supporting this, studies conducted in both Iran and Portugal reported the smallest service quality gap in the tangibles domain [1, 105], However, contrasting findings were observed in studies from Uganda and Saudi Arabia, where tangibles were associated with the highest gap [95, 99], suggesting that contextual and infrastructural differences may influence patient evaluations of this domain.

Following the implementation of the HTP, a reduction of 0.176 in the quality gap was observed for inpatient services, while outpatient and PHC services experienced increases of 0.02 and 0.24, respectively. Concurrently, negative quality gaps of 0.68 (inpatient), 0.78 (outpatient), and 1.18 (PHC) were recorded, indicating that despite modest relative improvements in some indicators, perceived service quality in all three sectors remained lower than users’ expectations. In line with this, a meta-analysis of studies conducted in Iran revealed negative gaps of 0.9, 0.48, and 0.83 for inpatient [30], outpatient [1], and PHC services [97].

Although the HTP improved hospital performance indicators such as bed occupancy and turnover rates, its focus on enhancing inpatient services in government hospitals was associated with a re-prioritization of public funds toward hospital-based care and unintended changes in financial protection mechanisms [12]. This approach contributed to the diminished prioritization of PHC and outpatient services, highlighting ongoing challenges in sustaining improvements and addressing persistent quality gaps in these sectors [106, 107]. The decline in PHC quality reflects the shift in resource allocation during the HTP, in which financial and workforce investments were disproportionately directed toward hospital-based services [108, 109]. This undermined PHC capacity by limiting operational budgets, reducing staffing stability, and constraining the system’s ability to provide continuous and equitable primary care. These consequences have direct implications for Iran’s UHC goals: unless PHC is re-established as a funding and service-delivery priority, through protected PHC budgets, workforce retention strategies, and strengthened referral and integration mechanisms, progress toward equitable and sustainable UHC may be compromised.

Beyond the direct effects of the HTP, several macro-level economic and social conditions in Iran may have acted as important confounding variables influencing patients’ expectations and perceptions of care. Sanctions and persistent inflation have increased healthcare costs, contributed to medicine shortages, and reduced access, particularly for vulnerable groups, leading to financial hardship, delayed or inadequate treatment, and declining trust in the health system. Likewise, reductions in public health expenditure and broader economic instability have resulted in staff shortages, outdated infrastructure, and inequitable service delivery, all of which can heighten expectations while simultaneously lowering perceived service quality. Some studies have also indicated that these economic and political constraints interact with evolving cultural norms and changing patient expectations, shaping how individuals evaluate the quality of services they receive. These contextual realities may partially explain why expectations increased more sharply than perceptions in our meta-analysis and why the service quality gap persisted despite the implementation of the HTP [18, 110, 111]. Moreover, the expansion of insurance coverage and the heightened visibility of the HTP in public discourse likely raised patients’ expectations faster than actual improvements in service delivery. Such policy signals may have strengthened perceptions of entitlement to better care even as structural limitations continued to restrict service performance.

Experiences from other health system reforms provide useful context for interpreting our findings. Countries such as Thailand [112] and Turkey [113] achieved measurable improvements in patient satisfaction and clinical quality largely because their reforms placed strong emphasis on strengthening primary healthcare, redistributing the workforce, and aligning payment incentives with PHC performance. In contrast, China’s 2009 reforms showed that prioritizing hospital-based investments can lead to gains in access but may leave PHC relatively underdeveloped, contributing to persistent service gaps [114], an outcome consistent with the widening quality gap observed in our post-HTP PHC results. Similarly, Mexico’s Seguro Popular demonstrated that expanding coverage alone does not guarantee improvements in service quality when provider incentives and PHC capacity remain weak [115]. Evidence from Rwanda further highlights that linking financial incentives to quality performance can effectively improve service delivery [116]. Taken together, these international experiences suggest that the limited improvements in perceptions and the widening quality gap in Iran may reflect an imbalance in resource allocation and incentive structures that favored hospital-based services over PHC during the HTP implementation.

## Study limitations

The present study was not without limitations. Primarily, the data analysis did not distinguish between the various groups (e.g., the elderly, pregnant women, etc.) and the different types of centers (e.g., government, private, military, etc.). In addition, more detailed subgroup analyses (e.g., by population characteristics or facility type) were not possible due to insufficient reporting in the primary studies. Another limitation of this study was the potential for publication bias and heterogeneity of findings. The substantial heterogeneity observed in the pooled estimates, largely due to differences in study settings, populations, measurement years, and SERVQUAL implementation, should also be acknowledged. In addition, the certainty of the evidence was not formally assessed using approaches such as GRADE, which should be considered when interpreting the pooled findings. Therefore, it is recommended that the reader consider this issue when interpreting the results of the study. Additionally, due to the relatively underdeveloped Iranian scientific database, some studies may have been overlooked in the review process. Finally, it should be acknowledged that other variables affecting the expectations and perceptions of patients, such as changes in economic, social and cultural conditions, etc., have not been controlled. Consequently, it cannot be definitively stated that the increase observed in the quality of service gap is a result of the implementation of HTP.

## Conclusion

This systematic review and meta-analysis indicates that although patients’ expectations and perceptions showed slight improvement after the implementation of the HTP in Iran, the overall quality gap widened. The largest deficits were observed in the dimensions of empathy and responsiveness, and the most notable deterioration occurred within primary healthcare services, while inpatient services demonstrated modest gains. These findings suggest that the reforms associated with the HTP may have benefited hospital-based services more than outpatient and primary healthcare settings.

To address these gaps, policymakers should prioritize strengthening primary healthcare through improved resource allocation, workforce support, and better organizational processes. Enhancing responsiveness and interpersonal aspects of care, particularly communication, empathy, and patient engagement, should also be incorporated into continuous professional development programs. Establishing routine patient-experience monitoring systems and integrating feedback into managerial decision-making can further support timely improvements in service quality. Future research should use longitudinal and interventional designs to more accurately assess the effects of targeted reforms across different healthcare levels and populations. Overall, aligning healthcare services with patient expectations requires a more balanced and patient-centered approach across the entire continuum of care.

## Data Availability

The data that support the findings of this study are available from the corresponding author.

## Abbreviations

HTP: Health transformation plan
PHC: Primary health care
WHO: World Health Organization
OECD: Organization for economic co-operation and development
UHC: Universal health coverage
LMICs: Low- and middle-income countries
OOP: Out-of-pocket
PRISMA: Preferred reporting items for systematic reviews and Meta-Analyses
SID: Scientific Information Database
MeSH: Medical subject headings
STROBE: Strengthening the reporting of observational studies in epidemiology
CMA: Comprehensive meta-analysis
SD: Standard deviation
CI: Confidence interval
I^2^: I-squared
